# Is This Stalking? Perceptions and Victimization Experiences of Stalking and Intrusive Behaviors in Hong Kong, Mainland China, and Ghana

**DOI:** 10.3390/ijerph19116689

**Published:** 2022-05-30

**Authors:** Heng Choon (Oliver) Chan

**Affiliations:** Teaching Laboratory for Forensics and Criminology, Department of Social and Behavioral Sciences, City University of Hong Kong, Hong Kong SAR, China; oliverchan.ss@cityu.edu.hk

**Keywords:** stalking, intrusive behavior, perception, experience, victimization, Hong Kong, Mainland China, Ghana

## Abstract

Many studies of stalking and intrusive behaviors are conducted with samples from individualist Western cultures, and limited information is available on such behavior in collectivist cultures. By using a sample of 1143 adults (440 males and 703 females) from Hong Kong (*n* = 305), mainland China (*n* = 464), and Ghana (*n* = 374), this study compares perceptions and experiences of stalking and intrusive behaviors as well as the frequency and duration of the participants’ worst experiences with such behaviors. The lifetime prevalence rate of stalking victimization for the overall sample was 34.6%, 22.3% for the Hong Kongers, 32.3% for the mainland Chinese, and 47.3% for the Ghanaians. Relative to the Hong Kongers and Ghanaians, the mainland Chinese were more likely to judge most intrusive activities as unacceptable. However, the mainland Chinese were generally less likely to have experienced the listed intrusive activities than their counterparts. The Ghanaians, in contrast, reported significantly more victimization experiences than the Hong Kongers and the mainland Chinese, especially with aggression and surveillance, unwanted attention, and persistent courtship and imposition types of behaviors. Furthermore, the mainland Chinese and Ghanaians generally reported significantly higher frequencies of stalking and intrusive behavior in their worst experiences than did the Hong Kongers. Conversely, the Hong Kongers and Ghanaians reported significantly more persistent types of stalking and intrusive behaviors than the mainland Chinese. The results of this study indicate the need for anti-stalking legislation in Hong Kong, mainland China, and Ghana, given the devastating nature and consequences of stalking and intrusive behaviors there.

## 1. Introduction

Stalking is a serious global public health and criminal justice problem that can have a devastating impact on victims, their families, and the wider community. Stalking is an old behavior but a relatively new offense: it was not legislated against until California enacted the world’s first anti-stalking laws in 1990 [1]. Hong Kong, mainland China, and Ghana have yet to implement anti-stalking laws. Perhaps the severity of impact has not been widely recognized in these societies. Stalking is difficult to define. Some behaviors that can be regarded as stalking are similar to behaviors thought to be acceptable within a courtship context. For example, behaviors such as making telephone calls, sending gifts, and waiting outside a person’s workplace may not seem threatening in isolation and within the context of courtship. However, if these behaviors are unwanted and executed repeatedly against the same person, they can be threatening [2,3,4]. No standardized definition or defining criteria of stalking have been established; rather, it has been defined in many ways, including by strict legal definitions that require the stalker to demonstrate intent and the victim to feel fear or by broader definitions that include lists of constituent behavior, see, e.g., [5,6]. Stalking commonly comprises a wide array of behaviors, ranging from mere harassment (e.g., text messaging, standing outside the victim’s home or workplace) to life-threatening behaviors (e.g., threatening to injure or murder the victim) [7,8].

Most research on stalking was conducted in the U.S., the U.K., and Australia. Prevalence rates of stalking victimization vary according to the definition of “stalking” that is applied. Large-scale, representative questionnaires conducted in these countries have found relatively similar lifetime prevalence rates: 1 in 5 women and 1 in 19 men in the U.S. [9], 1 in 5 women and 1 in 18 men in the U.K. [10], and 1 in 5 women and 1 in 12 men in Australia [11] have experienced being stalked. Notably, recent research has consistently demonstrated that stalking and intrusive incidents are not unusual and possibly occur in every country [12,13,14]. An increasing number of empirical studies conducted with samples from under-researched populations in recent years (e.g., Denmark, Finland, Germany, Ghana, Hong Kong, Lithuania, mainland China, the Netherlands, Portugal, South Africa, Singapore, and Spain) found that the prevalence rates of stalking and intrusive incidents to range from 5% to 55% [7,12,13,14,15,16].

Regardless of the type and severity of stalking and intrusive activities, the adverse effects experienced by the victims are clearly substantial. Stalking victims commonly experience a large range of psychological, physical, social, occupational, and financial costs [3]. Stalking victimization can result in increased levels of stress, fear, helplessness, and disenchantment [17]. Notably, persistence in stalking may result in serious psychological damage to the victims. Victims’ health risks from stalking are also influenced by the coping approaches that they adopt (e.g., avoidant, proactive, passive, compliant, and aggressive strategies) [18,19]. A large majority of those who perpetrate stalking and intrusive behaviors (i.e., stalkers) are the victims’ former intimate partners (49–81%), followed in frequency by victims’ acquaintances (13–22.5%) and strangers (10–18%) [20,21,22,23]. Stalkers were found to be motivated by a desire to control their victims or to rebuild a relationship with them (mostly by former intimate partners), by the victim’s attractiveness (mostly by acquaintances), or by a desire to harass or harm the victim (e.g., victim intimidation; mostly by strangers) [3,20].

In addition to studies on stalkers and victims of stalking, an increasing number of studies were conducted on laypersons’ perceptions of stalking. Empirical studies on perceptions of stalking have primarily sought to identify and characterize behaviors that the public regards as stalking and intrusive behavior [7,16,24,25,26]. Empirical research on public perceptions of stalking can elucidate the extent to which the public is aware of the nature and adverse consequences of stalking incidents. More importantly, this line of research can help to address misconceptions that the public may hold about stalking and intrusive behavior that contribute to a lack of demand for necessary social and policy change.

Research on public perceptions of stalking is especially relevant in jurisdictions that have not outlawed stalking activities, such as Hong Kong, mainland China, and Ghana. Thus, this exploratory study was undertaken to determine whether there are geographical differences in perceptions and experiences of stalking victimization. The data were collected from a survey of university students in Hong Kong, mainland China, and Ghana regarding their perceptions and victimization experiences of stalking and intrusive behaviors as well as the frequency and duration of their worst stalking victimization experiences. To date, only six such empirical studies were conducted with samples recruited from Hong Kong, mainland China, and Ghana (i.e., perceptions of stalking behavior in Hong Kong and mainland China [7], psychosocial characteristics of stalking victims in Hong Kong [15], perceptions and experiences of stalking victimization in Ghana [16], stalking victim coping strategies in Hong Kong [18], stalker–victim relationships in Hong Kong [20], stalking perpetration behaviors and motives in Hong Kong [27]). Given the scarcity of research into the perceptions and experiences of stalking victimization in these areas, this study is important not only to advance our knowledge of stalking behavior but also to fill a gap in the literature on geographical diversity by drawing from under-researched populations.

### 1.1. Cross-Cultural Research on Stalking

According to Hofstede’s [28] original cultural framework, the cultural value dimension of the individualism–collectivism spectrum is defined as “the degree of which people in a country prefer to act as individuals rather than as members of groups” (p. 6) [29]. More specifically, individualism is “a loosely knit social framework in which people are supposed to take care of themselves and of their immediate families only”, while collectivism “is characterized by a tight social framework in which people distinguish between ingroups and outgroups, they expect their ingroup to look after them, and in exchange for that they feel they owe absolute loyalty to it” (p. 45) [30]. The scores range from 1 for the lowest (extreme end on collectivism) to 100 for the highest (extreme end on individualism). Although there is an abundance of literature on perceptions of stalking, most of these studies were conducted within individualistic cultures. The most recent Hofstede individualism–collectivism scores of these three countries are 91 (the U.S.), 90 (Australia), and 89 (the U.K.) (The Hofstede individualism–collectivism scores were accessed on 23 March 2022 from https://www.hofstede-insights.com/country-comparison/). Hong Kong, mainland China, and Ghana are culturally collective societies, with Hofstede’s individualism–collectivism scores of 25, 20, and 15, respectively. As Chapman and Spitzberg [31] argued, the findings of stalking studies with samples from individualist cultures cannot be generalized to collectivist cultures. Hence, the findings of the present study can advance our knowledge on the topic.

A limited number of studies made cross-cultural comparisons of perceptions and experiences of stalking. In their study of 143 American and 233 Japanese university students, Chapman and Spitzberg [31] reported that more American (41%) than Japanese (34%) participants who had been “persistently pursued” were inclined to believe that their experience constituted stalking. In contrast, significantly more Japanese participants (40%) perceived their experience as “threatening” than their American counterparts (11%), and this trend was more prevalent in males. Chapman and Spitzberg [31] attributed these differences in part to the collectivist nature of Japanese society and the individualist nature of American society. More recently, in a study of 1734 female university students from 12 countries (Armenia, Australia, England, Egypt, Finland, India, Indonesia, Italy, Japan, Portugal, Scotland, and Trinidad), Sheridan et al. [13] adopted Hofstede’s dimensions of national cultures [32] and the Gender Empowerment Measure (GEM; used to measure gender inequality and females’ relative empowerment between countries) to study cross-cultural differences in experiences of stalking victimization. Females from countries scoring lower on individualism (e.g., Indonesia, Trinidad) reported more severe intrusions (e.g., forced sexual contact, being spied upon), while females from countries with higher individualism scores (e.g., Finland, Scotland) reported more innocuous intrusions (e.g., being asked for dates, being asked for casual sex at social events). Moreover, the GEM and individualism–collectivism scores were significantly correlated (0.60), with lower gender equality ratings associated with higher collectivism scores and vice versa.

Sheridan et al. [14] conducted another study on perceptions and victimization experiences of stalking and intrusive activities that compared subcultures within a single country. In their study of 89 Chinese, 69 Indian, and 68 Malay females in Singapore, they found minimal differences in courtship behaviors. The authors suggested that the overarching national attitudes toward women had more influence than subcultural variation in determining which types of intrusive behaviors the participants were likely to have experienced. In a more recent study by Chan and Sheridan [7] comparing the perceptions of stalking in a sample of 1846 university students from Hong Kong and mainland China, they found that although a significantly larger proportion of mainland Chinese than Hong Kongers deemed some of the intrusive activities to constitute stalking (e.g., making the victim fearful for their safety or life, threatening to harm or kill the victim, vandalizing the victim’s property or damaging something the victim valued, and sending unsolicited or harassing emails to the victim), the effect sizes for these differences were small. Chan and Sheridan [7] reasoned that this result may be explained by a high degree of similarity between the two cultures examined or perhaps by the fact that the mainland Chinese sample recruited in Hong Kong had adapted psychologically to the local culture and lifestyle (i.e., acculturated). It is possible that mainland Chinese in their home country may differ in their perceptions of stalking, which was found in this study.

### 1.2. Present Study

This study focuses on perceptions and victimization experiences of stalking and intrusive behaviors in Hong Kong, mainland China, and Ghana. Hong Kong has been a semi-autonomous city (a special administrative region [SAR]) of the People’s Republic of China (PRC) since 1 July 1997. Prior, Hong Kong was a British colony for more than 150 years. Geographically situated in the East Asia region, Hong Kong is regarded as a major regional financial hub. As of 2021, it had a population of 7.41 million [33], with approximately 95% of its occupants being of Chinese descent. The official languages in Hong Kong are English and Chinese Cantonese. As a modern Chinese society with substantial Western influences, Hong Kongers largely balance a modern Western lifestyle with traditional Chinese cultural values and practices. Traditional Chinese culture can be traced back over 4000 years, during which time the same language has been maintained. Traditional Chinese culture consists of diverse and often competing schools of thought, including Confucianism, Taoism, and Buddhism. Confucianism, which forms the foundation of the Chinese cultural tradition, emphasizes human relationships, social structures, virtuous behavior, and work ethic [34]. The basic teachings of Confucius focus on the Five Constant Virtues, namely humanity, righteousness, propriety, wisdom, and faithfulness. This further describes the five basic human relations and principles for each relation, namely love and obedience, loyalty and duty, seniority and modeling subject, obligation and submission, and trust [35]. Relationships are formed to ensure a harmonious society that emphasizes the significance of loyalty and filial piety. This cultural value system provides Chinese people with their basic identity. The Hong Kong legal system adopts the British common law system, which primarily stresses the rule of law and due process [36]. Relevant to this study, stalking has not been legislated against in Hong Kong.

Mainland China, also commonly referred to as the PRC, is the most populous country in the world, with a population of 1.41 billion in 2020 [37]. Mainland China consists of 31 provinces, autonomous regions, and municipalities. According to the 2020 census, the majority of the population is Han-Chinese (91.11%), and the remaining 8.89% comprises 55 minority ethnic groups (e.g., Zhuang, Hui, Manchu, Uyghur, Miao, Yi, Tujia, Tibetan, and Mongol). Most of the minority ethnic groups are concentrated near the country’s northwestern, northern, northeastern, southern, and southwestern borders, although some minorities reside in the central areas of the mainland. The official language in mainland China is Chinese Mandarin (also known as Putonghua). People in mainland China generally adhere to traditional Chinese teachings and cultural values. Nonetheless, Westernized beliefs and practices have also been observed in some of China’s megacities (e.g., Beijing, Shanghai, Guangzhou, and Shenzhen) in recent decades. Mainland China has a socialist legal system (a civil law system) with Chinese characteristics. As in Hong Kong, there is no anti-stalking legislation in mainland China.

Ghana, formerly known as the Gold Coast because of its abundance of gold, is a West African state sharing borders with Cote d’Ivoire to the west, Burkina Faso to the north, and Togo to the east, and the Gulf of Guinea and the Atlantic Ocean to the south. Its population was 30.83 million in 2021 [38], and the majority of its population is Akan (47.5%), the remainder comprising Mole-Dagbon (16.6%), Ewe (13.9%), Ga-Dangme (7.4%), Gurma (5.7%), Guan (3.7%), Manda (1.1%), and other ethnic groups (1.4%). Ghana was the first sub-Saharan country to declare independence from European colonization, which occurred on 6 March 1957 [36]. Given its historical ties, Ghana’s official language is English, and its legislation and judicial practices are heavily influenced by the British system, although the Ghanaian criminal justice system has experienced significant changes since the country’s independence [39]. Like many African countries, Ghana has not yet legislated against stalking and has no formalized criminal justice response to stalking.

In this study, “stalking” is defined as “a series of acts directed at a specific person that, taken together over a period of time, cause him (or her) to feel harassed, alarmed, or distressed” [40]. This exploratory study is particularly important for two reasons. First, it is the first empirical study to compare perceptions and victimization experiences of stalking and intrusive behaviors among samples of Hong Kongers, mainland Chinese, and Ghanaians. Next, this study explored the frequency and duration of the participants’ worst experiences of stalking and intrusive behaviors. Importantly, this study adds geographical diversity to the literature on stalking, as it draws from samples in under-researched populations. As the sample in this study was recruited from collective societies, it was expected that the cultural differences between the participants would not be great. Perhaps more importantly, the findings of this study contribute to the repertoire of the literature and inform practices in the areas of improving assistance strategies for victims of stalking and developing or refining public and social policies to help curb the incidents of stalking perpetration from in the collectivistic culture context. As many countries in Asia and Africa are collectivistic societies, this study can be particularly important and contribute to the stalking literature, given most of the stalking research was conducted with samples from individualistic societies (e.g., the U.S., the U.K., and Australia). In view of the paucity of evidence on this subject, no directional hypotheses can be proposed.

## 2. Materials and Methods

### 2.1. Participants and Procedure

Ethical approval was obtained from the author’s institution prior to data collection. A total of 1143 participants aged 18 years and older were recruited from Hong Kong, mainland China, and Ghana for the study. The subsamples of 305 participants in Hong Kong (26.7%), 464 participants in mainland China (40.6%), and 374 participants in Ghana (32.7%) were recruited from one public university in each country/territory. About 65% of the participants were recruited at random from areas within the university campuses (e.g., reading corners, libraries, common areas, and cafeterias), and the remaining 35% were recruited through convenience sampling (e.g., recruitment from classrooms with prior consent from the instructors and via word of mouth among university students). The participants were provided the option of either completing the questionnaire online (i.e., Qualtrics Survey; about 80%) or on paper (about 20%). Informed consent was received from the participants, who were assured that their anonymous questionnaire responses would be kept confidential and used only for research purposes. Their participation in the study was voluntary without any monetary incentive, and no coercion was involved (e.g., potential consequences of nonparticipation to their academic performance). The average time required to complete the questionnaire was about 30 min, and the participation rate was approximately 90%.

The participants were 61.5% females (*n* = 703) and 38.5% males (*n* = 440), with a mean age of 22.19 years (*SD* = 3.57, range = 18–54). The Hong Kong subsample consisted of 80.3% females (*n* = 245) and 19.7% males (*n* = 60) with an average age of 20.16 years (*SD* = 1.39, range = 18–25). The subsample from mainland China comprised 57.8% females (*n* = 268) and 42.2% males (*n* = 196) with an average age of 21.95 years (*SD* = 2.32, range = 18–29). Finally, the subsample from Ghana included 50.8% females (*n* = 190) and 49.2% males (*n* = 184) who were aged 24.14 years on average (*SD* = 4.86, range = 18–54). Over two thirds of the total sample (67.8%) reported that they were single (76.4% of the Hong Kongers, 66.8% of the mainland Chinese, and 61.9% of the Ghanaians were single).

### 2.2. Measures

The modified version of the “Stalking: International perceptions and prevalence” questionnaire (SIPPQ) developed by Sheridan et al. [41] was used in this study. The original and modified versions of the measures (consisting of 42 and 47 stalking and intrusive behaviors, respectively) were adopted in at least 10 other studies [13,14,16], with samples recruited in 14 countries (Armenia, Australia, Egypt, England, Finland, Ghana, India, Indonesia, Italy, Japan, Portugal, Scotland, Singapore, and Trinidad). The samples collected were from a mix of community and university students, but none were regarded as representative of the wider population.

In addition to items on demographic characteristics (e.g., age, sex, and marital status), the SIPPQ includes three sections, the first of which measures the participants’ perceptions of a list of stalking and intrusive behaviors, some (but not all) of which are generally considered as constituting stalking. The participants were asked to read through a list of 47 stalking and intrusive behaviors and to indicate those they considered to be unacceptable from the perspective of the target of the behavior. A number of these items can also be found in two commonly adopted stalking measures, i.e., the Unwanted Pursuit Behavior Inventory (UPBI) [42] and the Obsessive Relational Intrusion scale (ORI-P) [43]. Sample items include “Following you”, “Asking you for a date repeatedly”, and “Intercepting mail/deliveries”. Some of the behaviors can be regarded as routine and innocuous acts, such as “A stranger engaging you in a conversation in a public place, such as a bus stop or in a café”. Based on the cluster analysis of the 47 behavioral items by Sheridan et al. [44] on 1734 young females from 12 countries, four clusters of stalking and intrusive behaviors emerged: (a) aggression and surveillance (19 items), (b) unwanted attention (7 items), (c) persistent courtship and impositions (9 items), and (d) courtship and information-seeking (10 items). Of note, two items did not load on any of the behavioral clusters. The Cronbach’s α of the measure in this study was 0.97 (Hong Kong = 0.89; mainland China = 0.98; Ghana = 0.89).

The second section consisted of a measure of individual experiences of stalking and intrusive behaviors. The participants read through the same list of 47 behavioral items, this time indicating those that they had personally experienced. The inclusion of innocuous behaviors rendered it unlikely that any participant had never experienced any of the listed activities. In the final section, a measure of the participants’ actual stalking and intrusive experiences, as opposed to a range of stalking and intrusive acts, was presented. Of note, to prevent potential priming effects, a definition of “stalking” was not presented to the participants. Instead, they were asked about their worst stalking or intrusive experience that was perpetrated by an individual. The participants were asked to read through a list of 15 behavioral items and indicate the frequency (0, 1–4, or 5+ times) of any they had been subjected to during their worst stalking or intrusive experience. Next, the participants were asked to report the duration of their worst stalking or intrusive experience with three options (“Less than two weeks”, “Two weeks to six months”, and “More than six months”).

The 15 behavioral items listed in the final section of SIPPQ were derived from Spitzberg and Cupach’s [3] meta-analysis that aimed to describe the behavioral content of stalking. Given that “stalking” has been defined as “a constellation of behaviors” (p. 1244) [45], this study acknowledges that the participants may have been subjected to activities other than the 15 behaviors listed. In order to classify an individual’s worst stalking or intrusive experience as stalking, this study used the operational criterion of “Experiencing any combination of the 15 behaviors on at least 10 occasions for a minimum of 2 weeks”. This criterion (i.e., at least a two-week period) was informed by the finding of Purcell et al. [2] that the lowest cut-off for intrusions to be perceived as problematic and by the widely used threshold of 10 occasions by Pathé et al. [46]. In this study, the “10 occasions” threshold was met by 10 or more instances of “1–4” occurrences and/or two or more instances of “5+” occurrences, and/or a combination of five or more “1–4” occurrences and one or more “5+” occurrences.

### 2.3. Data Analysis Plan

Descriptive statistics were calculated to identify the participants’ perceptions and victimization experiences of stalking and intrusive behaviors, the frequency of their worst victimization experiences, and the duration of their perceived worst victimization experiences. In addition, cross-tabular (i.e., chi-square or χ^2^) analyses were used to explore potential regional differences in the participants’ perceptions and victimization experiences of stalking and intrusive behaviors. Adopting Cohen’s standards for cross-tabular effect size interpretation, a measure of association (i.e., Cramer’s *V* coefficient (found between two variables with at least three levels on one variable)) was used to interpret the strength of the relationships between the two variables and, more importantly, to detect meaningful patterns. In chi-square analyses with two degrees of freedom (a 2 × 3 matrix), Cramer’s *V* values of 0.16 and below were regarded as weak, values between 0.17 and 0.28 were considered moderate, and values of 0.29 and above were considered strong effects [47]. The significance level was set at 0.05.

### 2.4. Ethical Considerations

This study was approved by the ethical review board of the author’s university. The participants could voluntarily end their participation, contact the primary investigator, and/or receive professional counseling at any time. The data were collected anonymously, with no personal identifying details recorded.

## 3. Results

### 3.1. Lifetime Prevalence of Stalking Victimization

According to the operational criterion of stalking victimization (i.e., experiencing any combination of the 15 behaviors on at least 10 occasions for a minimum of two weeks), 34.6% of the overall sample was found to have experienced an episode of stalking victimization (χ^2^ = 223.36, *p* < 0.001, Phi = 0.44; 34.1% males and 34.9% females). Breaking the results down by subsample, the lifetime prevalence rate of the Hong Kongers was 22.3% (χ^2^ = 61.82, *p* < 0.001, Phi = 0.45; 13.3% males and 24.5% females), the mainland Chinese rate was 32.3% (χ^2^ = 78.30, *p* < 0.001, Phi = 0.41; 35.2% males and 30.2% females), and the rate for Ghanaians was 47.3% (χ^2^ = 72.01, *p* < 0.001, Phi = 0.44; 39.7% males and 54.7% females).

### 3.2. Geographical Distribution of Perceptions of Stalking and Intrusive Behaviors

Table 1 presents the participants’ ratings of stalking and intrusive behaviors in Hong Kong, mainland China, and Ghana. Overall, more than 60% of the participants agreed that 31 of the 47 items (in four behavioral clusters) described unacceptable behaviors. The behaviors that were most frequently regarded as unacceptable were “Making death threats” (94.0%), “Criminal damage/vandalism to your property” (92.6%), and “Threatening to kill or hurt herself/himself if you refused to go out on a date with her/him” (92.0%). The behaviors that were least frequently considered to be unacceptable were “Asking you out ‘as just friends’” (42.7%), “Sending or giving you gifts” (43.6%), and “Seeing him/her at the same time each day” (47.3%).

Breaking the responses down by region, the behaviors that were most often regarded as unacceptable by the Hong Kongers were “Making death threats” (93.1%) and “Threatening to kill or hurt herself/himself if you refused to go out on a date with her/him” (92.1%); for the mainland Chinese, these were “Secretly taking your belongings” (98.3%), “Forced sexual contact” (97.8%), “Verbally abusing you” (97.8%), and “Multiple telephone calls which you don’t want to receive” (97.8%); and for the Ghanaians, they were “Making death threats” (90.6%) and “Criminal damage/vandalism to your property” (89.5%). The behaviors that were least commonly considered to be unacceptable by the Hong Kongers were “Sending or giving you gifts” (8.6%) and “Asking you out ‘as just friends’” (8.9%); for the mainland Chinese, they were “Sending you unwanted letters, notes, e-mail, or other written communications” (86.3%) and “Sending or giving you gifts” (91.6%); and for the Ghanaians, they were “Asking you out ‘as just friends’” (8.3%) and “Sending or giving you gifts” (12.6%).

It is interesting to note that significant differences between the participants’ geographic locations in perceptions of stalking and intrusive behaviors were observed for all 47 items. The mainland Chinese participants were significantly more likely than the participants from Hong Kong and Ghana to perceive the behaviors as stalking and intrusive behaviors. The effect sizes of these differences ranged from weak to strong (their Cramer’s *V* values ranged from 0.12 to 0.83; there were weak effects in 2 items, moderate effects in 8 items, and strong effects in 37 items). The relationships between items within the aggression and surveillance cluster ranged from weak to strong (Cramer’s *V* values ranging from 0.12 to 0.42); in the unwanted attention cluster, the relationships between items were strong (Cramer’s *V* values ranging from 0.42 to 0.64); in the persistent courtship and impositions clusters, the relationships were moderate to strong (Cramer’s *V* values ranging from 0.22 to 0.80); and in the courtship and information-seeking clusters, the relationships were strong (Cramer’s *V* values ranged from 0.61 to 0.83).

### 3.3. Geographical Distribution of Victimization Experiences of Stalking and Intrusive Behaviors

Table 2 shows the lifetime victimization experiences of stalking and intrusive behaviors among the Hong Kong, mainland Chinese, and Ghanaian subsamples. Of the 47 items, “Taking photographs of you without your knowledge” (45.6%), “Sending or giving you gifts” (42.3%), and “Doing unrequested favors for you” (42.1%) were the most commonly reported behaviors experienced by all of the participants, while “‘Outstaying his/her welcome’ in your home” (27.9%), “Spying on you” (28.5%), and “A stranger offering to buy you a drink in a café, restaurant, or bar” (29.6%) were the behaviors reported the least often.

These results are broken down by region as follows: the behaviors that were most often reported among the Hong Kongers were “A stranger engaging you in a conversation in a public place (e.g., at a bus stop or in a café)” (72.3%) and “Asking your friends, family, school, or work colleagues about you” (54.5%); among the mainland Chinese, they were “Doing unrequested favors for you” (32.3%) and “Sending you unwanted letters, notes, email, or other written communications” (31.0%); and among the Ghanaians, they were “Harming you physically” (89.3%) and “Criminal damage/vandalism to your property” (89.3%). The behaviors that were least often reported by the Hong Kongers were “Threatening to physically hurt you” (4.0%) and “Forced sexual contact” (4.3%); among the mainland Chinese, these were “Giving or sending you strange parcels” (8.2%) and “Driving, riding, or walking purposefully past your residence, school, or workplace” (8.8%); and among the Ghanaians, they were “A stranger engaging you in a conversation in a public place (e.g., at a bus stop or in a café)” (28.6%) and “Asking your friends, family, school, or work colleagues about you” (46.4%).

A comparison of victimization experiences of stalking or intrusive behaviors among the participants from Hong Kong, mainland China, and Ghana revealed significant differences in all of the items. The Ghanaian participants were significantly more likely than the Hong Kong and mainland Chinese participants to have experienced 44 of the listed stalking and intrusive behaviors, while the remaining three behaviors were significantly more frequently experienced by the Hong Kongers than the mainland Chinese and Ghanaians. The effect sizes of these differences were moderate to strong (Cramer’s *V* values ranging from 0.19 to 0.77; moderate effects in 11 items and strong effects in 36 items). Within behavioral clusters, the strength of items’ relationships was also noted to be moderate to strong (Cramer’s *V* values ranging from 0.23 to 0.78 for the aggression and surveillance cluster items, 0.21 to 0.73 for the unwanted attention cluster items, 0.28 to 0.71 for the persistent courtship and impositions cluster items, and 0.19 to 0.62 for the courtship and information-seeking cluster items).

### 3.4. Geographical Distribution of Perceived Worst Experiences of Stalking and Intrusive Behavior Victimization Frequency

The frequencies of the participants’ reported worst victimization experiences of stalking and intrusive behaviors are shown in Table 3. Among the 15 listed items, the behaviors most frequently reported were “Phone calls, text messages, gifts, or letters” (31.8% of the participants had experienced this 5+ times from the same individual), followed by “Emails and/or messages on social media (e.g., Facebook) and other web-based communications” (23.3% reported 5+ experiences) and “Declaring love for you” (22.9% reported 5+ experiences). The behaviors least reported were “Threatening to hurt you” (3.5% reported 5+ experiences), “Actually hurting people you care about” (3.5% reported 5+ experiences), and “Damage to your property, wrecking things you care about, or hurting your pet” (3.9% reported 5+ experiences).

Delving into the results according to the participants’ countries and territories, the three most often-reported worst stalking and intrusive experiences among the Hong Kongers, mainland Chinese, and Ghanaians were “Phone calls, text messages, gifts, or letters” (28.0%, 22.4%, and 44.2% reported 5+ experiences, respectively), “Emails and/or messages on social media (e.g., Facebook) and other web-based communications” (17.0%, 25.2%, and 27.1% reported 5+ experiences, respectively), and “Declaring love for you” (8.9%, 18.9%, and 39.0% reported 5+ experiences, respectively). The behaviors that were least often regarded as their worst stalking and intrusive experiences among the Hong Kongers were “Trespassing on your property” (0.4% reported 5+ experiences) and “Threatening to hurting people you care about” (0.4%); among the mainland Chinese, these were “Actually hurting people you care about” (5.9%) and “Threatening to hurt you” (6.2%); and for the Ghanaians, they were “Damage to your property, wrecking things you care about, or hurting your pet” (1.4%) and “Threatening to hurting people you care about” (2.8%).

In a comparison of the results by region, significant differences were observed in all 15 items. Relative to the other participants, the mainland Chinese reported significantly more 5+ incidents of eight of the listed stalking and intrusive behaviors, while the Ghanaians had experienced more 5+ incidents of stalking and intrusive behavior victimization involving seven of the behaviors. However, the strengths of these relationships were weak to moderate (Cramer’s *V* values ranging from 0.07 to 0.24).

### 3.5. Geographical Distribution of Perceived Worst Victimization Experience of Stalking and Intrusive Behavior Duration

The findings on the duration of the participants’ perceived worst victimization experiences of stalking and intrusive behaviors are presented in Table 4. In general, the behaviors that the participants most often experienced for longer periods were “Phone calls, text messages, gifts, or letters” (29.3% persisted for more than 6 months), “Declaring love for you” (28.9%), and “Emails and/or messages on social media (e.g., Facebook) and other web-based communications (22.4%). The least persistent behaviors reported by the participants were “Damage to your property, wrecking things you care about, or hurting your pet” (5.4%), “Threatening to hurt you” (5.6%), and “Threatening to hurt people you care about” (5.8%).

More specifically, the behaviors most commonly experienced for longer periods by the Hong Kongers were “Physically and/or sexually attacking you” (25.0% persisted for more than 6 months) and “Actually hurting people you care about (23.1%); among mainland Chinese, these were “Phone calls, text messages, gifts, or letters” (16.0%) and “Emails and/or messages on social media (e.g., Facebook) and other web-based communications” (15.7%); and among the Ghanaians, they were “Phone calls, text messages, gifts, or letters” (46.8%) and “Declaring love for you” (43.8%). The behaviors least frequently experienced for longer periods by the Hong Kongers were “Threatening to hurt people you care about” (0.0%) and “Getting other people to engage in any of the 14 behaviors listed above on their behalf” (8.8%); among the mainland Chinese, they were “Trespassing on your property” (7.3%) and “Threatening to or actually hurting him/herself” (8.8%); and among the Ghanaians, they were “Damage to your property, wrecking things you care about, or hurting your pet” (2.4%) and “Actually hurting people you care about” (3.5%).

Significant country/territorial differences were found in nine stalking and intrusive behaviors. Of these nine items, the Hong Kongers and Ghanaians reported significantly higher frequencies (persisting for more than 6 months) than their counterparts on four items each, while the mainland Chinese only reported significantly higher frequencies than the others on one item. The strengths of association in these significant differences were weak to moderate, ranging from 0.13 to 0.27.

## 4. Discussion

Stalking victimization is a global concern with adverse effects on the victims’ physical, psychological, emotional, and cognitive well-being. This study is important not only in its general contribution to the knowledge of public perceptions of stalking and intrusive behavior but also for its less commonly researched population—that of Hong Kongers, mainland Chinese, and Ghanaians. By using a large sample of 1143 adults in Hong Kong, mainland China, and Ghana, this study explored geographical differences in individuals’ perceptions concerning stalking and intrusive behavior, their experiences in stalking victimization, and the frequency and duration of their worst stalking victimization experiences. The overall lifetime prevalence of stalking victimization in this study was 34.6%, with a higher rate reported by females than by males (34.9% vs. 34.1%). In terms of the study’s geographical subsamples, the lifetime prevalence rates of the Hong Kongers, mainland Chinese, and Ghanaians were 22.3% (13.3% males and 24.5% females), 32.3% (35.2% males and 30.2% females), and 47.3% (39.7% males and 54.7% females), respectively. With the exception of the mainland Chinese males, the gendered trend of prevalence found in this study was in line with the literature, with a higher prevalence estimate found in females than in males. The meta-analysis of 103 stalking studies performed by Spitzberg [22] estimated prevalence rates of 23.5% for females and 10.5% for males. Of note, the prevalence rate in this study was higher than the reported mean incidence rate of 19% in college population studies [22]. This difference may be due to the form of measurement used, as the present study did not require the victim to report fear or threat. Furthermore, Purcell et al. [48] noted that studies that determine stalking through the presentation of behavioral items seem to generate higher prevalence rates than those that use a single rating question (e.g., “Have you been stalked during the past 12 months?”).

Several noteworthy trends in geographical differences emerged in this study that warrant further discussion. With at least 70% of the participants perceiving an activity as unacceptable, all of the listed activities in the aggression and surveillance cluster were the most perceived by the participants to constitute stalking (100%; 100% by the mainland Chinese, 84.2% by the Hong Kongers, and 73.7% by the Ghanaians), followed by the items in the persistent courtship and impositions cluster (33.3%; 100% by the mainland Chinese, 22.2% by the Ghanaians, and 11.1% by the Hong Kongers). Conversely, with 50% as the upper limit, activities related to courtship and information-seeking were the least likely to be perceived by the participants to constitute stalking (40%; 100% by the Hong Kongers and Ghanaians and 0% by the mainland Chinese). In terms of the participants’ experiences of stalking victimization, activities in the aggression and surveillance cluster were the most frequently experienced by the participants (28.5–45.6%; 47.6–89.3% by the Ghanaians, 4–50.8% by the Hong Kongers, and 11.4–28% by the mainland Chinese), followed by activities in the courtship and information-seeking cluster (31.9–42.1%; 28.6–78.6% by the Ghanaians, 19.1–72.3% by the Hong Kongers, and 12.5–32.3% by the mainland Chinese) and the persistent courtship and impositions cluster (27.9–42.3%; 59.4–86.1% by the Ghanaians, 8.7–43.8% by the Hong Kongers, and 10.8–26.9% by the mainland Chinese). Table 5 presents the comparison of perceptions and experiences of stalking victimization of the Hong Kongers, mainland Chinese, and Ghanaians.

An interesting trend was observed: the mainland Chinese participants were more likely to judge the listed activities to be unacceptable (a higher rate of perception) but were less likely to have personally experienced them (a lower rate of experience). This observation can be explained from the perspective of traditional Chinese norms and practices. Social control and social stability in traditional Chinese societies have relied primarily on morals rather than laws. Deeply influenced by Confucianism, the mainland Chinese have long displayed a marked abhorrence of formal law and have instead relied on moral codes and the exemplary behavior of authority figures to maintain social order and prevent criminal activity [49,50]. In simple terms, formal social control is imposed by law and enforced by official controlling organizations (e.g., police), while informal social control is based on morality and performed by unofficial controlling groups or individuals (e.g., parents, teachers). Traditional Chinese societies are mostly group- and family-based. In collectivist mainland China, family members are expected to understand the moral code and to know one another well, creating the conditions for effective informal control in traditional Chinese families. Hence, it is not surprising that mainland Chinese lean heavily on moral teachings to judge activities that may seem intimidating as socially inappropriate and that moral-based, informal control is effective in deterring them from engaging in socially unacceptable behavior (e.g., stalking and intrusive behavior).

Owing to a demographic transformation in Ghana, a rapid shift of the population from rural to urban areas in recent decades has resulted in numerous social problems, such as unemployment (especially youth unemployment) and poor municipal infrastructure and services (e.g., security services) [51]. Similar to many traditional Asian societies, Ghanaian society is a largely patriarchal society, especially in rural areas. In a patriarchal society, women are generally subordinate to men in nearly every social domain. Official police reports reveal a growing incidence of urban and interpersonal violent crimes (e.g., assault, murder, armed robbery, and theft) in Ghana, especially against women and girls, which has increased its citizens’ fear of crime [52,53]. This is evident in this study’s finding of a higher incidence rate of aggressive behavior (Cluster 1; e.g., “Harming you physically”, “Criminal damage/vandalism to your property”, and “Forced sexual contact”) reported by the Ghanaian participants than by the Hong Kongers and mainland Chinese.

This study also investigated the frequency and duration of the participants’ perceived worst experiences of stalking and intrusive behaviors. In general, the mainland Chinese participants reported having experienced significantly more (i.e., over five times more) instances of stalking and intrusive behaviors composing their worst experiences (eight activities, e.g., “Ruining your reputation” (12.6%), “Threatening to hurt people you care about” (8.4%), and “Threatening to or actually hurting him/herself” (8.3%)), followed by the Ghanaians (seven activities; e.g., “Phone calls, text messages, gifts, or letters” (44.2%), “Declaring love for you” (39%), and “Emails and/or messages on social media and other web-based communications” (27.1%)). Conversely, the Ghanaians and Hong Kongers reported having experienced significantly longer durations (i.e., more than 6 months) of more activities. Specifically, they each reported four activities as the worst experiences. The Ghanaians reported, e.g., “Phone calls, text messages, gifts, or letters” (46.8%), “Declaring love for you” (43.8%), and “Emails and/or messages on social media and other web-based communications” (30.6%). The Hong Kongers reported, e.g., “Physically and/or sexually attacking you” (25%), “Actually hurting people you care about” (23.1%), and “Damage to your property, wrecking things you care about, or hurting your pet” (18.2%).

Irrespective of the study populations, studies consistently found that the longer the stalking victimization persists, the greater the potential for psychological, physical, social, and emotional harm to the victims. For example, Kamphuis et al. [54] found a positive relationship between stalking duration and post-traumatic stress symptoms. Moreover, the stalker–victim relationship was reported to be a good predictor of stalking duration, with rejected, former intimate partners being the most persistent and strangers being the least persistent [2,55]. Rosenfeld [56] found that former intimate partners, particularly those diagnosed with personality disorders, are more likely to be arrested for further stalking offenses. Thus, it is important to intervene in any persistent stalking episode promptly before it escalates to more serious offenses (e.g., sexual assault, rape, homicide). Among all of the victim coping approaches, the proactive coping approach (i.e., actively seeking formal and informal social support to put a stop to the stalking) is arguably the most effective [18,19].

### Limitations of the Study

The findings of this study should be interpreted cautiously in view of several limitations. First, this study was limited by the use of self-reported data and a lack of depth in the participants’ responses regarding their perceptions and victimization experiences. For example, the SIPPQ does not assess the number of times that the 47 behaviors were experienced. As stalking behavior is commonly characterized by repetition and persistence of behavior, future studies may consider including a frequency- and severity-based determination of unacceptability. Moreover, biases such as retrospective recall bias and social desirability may have affected the participants’ truthfulness when reporting their experiences, leading to the possible under-reporting of their victimization. It is also noteworthy that the Western-developed measures used in this study have not been culturally validated. Hence, the validity of these measures used in non-Western samples (e.g., Asian and African samples) remains unclear. Future research could incorporate a measure for response bias to minimize participants’ potential reporting biases, use measures specific to studied cultures (e.g., collectivist cultures), and explore additional victim characteristics in conjunction with other offending and circumstantial factors to obtain more comprehensive information on the stalking victimization experience. This can possibly be performed through in-depth follow-up interviews. Furthermore, the sampling population was limited to small and non-random samples recruited from universities in Hong Kong, mainland China, and Ghana. Therefore, the findings cannot be generalized to their wider populations. In order to address this limitation, future studies could recruit a larger sample size and participants from all walks of life. Such effort could help to explore if stalking behavior is more prevalent among younger populations or also frequently observed among individuals in older age groups.

## 5. Conclusions

Notwithstanding its limitations, this study is the first empirical research to compare the perceptions and victimization experiences of stalking and intrusive behaviors with samples from Hong Kong, mainland China, and Ghana. The strengths of this study are clear. First, it has taken an important step toward a better understanding of the perceptions and experiences of stalking victimization in collectivist cultures (e.g., Asian and African cultures). Second, the findings of this study further point to the universality of stalking and intrusive behavior in that the findings support those of studies conducted in individualistic cultures (e.g., the U.S., the U.K., Australia). Given the gravity of stalking victimization and its potential for escalation into violence (e.g., sexual assault, rape, homicide), it is of the utmost importance that specific anti-stalking legislation is introduced in jurisdictions where it does not presently exist. Furthermore, it is essential to provide prompt and appropriate intervention to stalking victims to reduce the probability of persistent victimization. Regardless of the interventional approaches they use, mental health professionals should not assume that what has been practiced and found effective in the West is applicable in a non-Western context. Any intervention strategies should be culturally sensitive to achieve the optimal effect.

## Figures and Tables

**Table 1:** Geographical distribution of perceptions of stalking and intrusive behaviors (*n* = 1143).

Items	Perceptions of Stalking and Intrusive Behaviors				Geographical Differences	
	Overall (*n* = 1143) *n* (%)	Hong Kong (*n* = 305) *n* (%)	Mainland China (*n* = 464) *n* (%)	Ghana (*n* = 374) *n* (%)	χ^2^	Cramer’s *V*
Cluster 1: Aggression and surveillance (19 items)						
01. Making death threats.	1074 (94.0%)	283 (93.1%)	452 (97.4%)	339 (90.6%)	17.63	0.12 ***
02. Criminal damage/vandalism to your property.	1057 (92.6%)	274 (90.1%)	449 (96.8%)	334 (89.5%)	19.64	0.13 ***
03. Threatening to kill or hurt herself/himself if you refused to go out on a date with her/him.	1051 (92.0%)	280 (92.1%)	452 (97.4%)	319 (85.3%)	41.48	0.19 ***
04. Forced sexual contact.	1035 (90.8%)	278 (91.7%)	454 (97.8%)	303 (81.2%)	68.69	0.25 ***
05. Harming you physically.	1035 (90.7%)	275 (90.8%)	449 (96.8%)	311 (83.2%)	45.53	0.20 ***
06. Verbally abusing you.	1035 (90.6%)	257 (84.5%)	454 (97.8%)	324 (86.6%)	48.77	0.21 ***
07. Threatening to physically hurt you.	1019 (89.4%)	268 (88.4%)	451 (97.2%)	300 (80.4%)	61.67	0.23 ***
08. Hurting you emotionally (verbal abuse, ruining your reputation).	1006 (88.2%)	244 (80.5%)	450 (97.0%)	312 (83.4%)	59.59	0.23 ***
09. Physically hurting someone you care about.	995 (87.2%)	253 (83.5%)	449 (96.8%)	293 (78.3%)	68.08	0.24 ***
10. Secretly taking your belongings.	979 (85.8%)	240 (79.2%)	456 (98.3%)	283 (75.7%)	101.61	0.30 ***
11. Trying to manipulate or force you into dating her/him.	967 (84.8%)	247 (81.5%)	451 (97.2%)	269 (71.9%)	105.68	0.30 ***
12. Spying on you.	960 (84.1%)	255 (84.2%)	453 (97.6%)	252 (67.4%)	141.97	0.35 ***
13. Trespassing on your property.	947 (83.0%)	223 (73.6%)	450 (97.0%)	274 (73.3%)	108.40	0.31 ***
14. Confining you against your will.	943 (82.6%)	208 (68.6%)	451 (97.2%)	284 (75.9%)	121.66	0.33 ***
15. Multiple telephone calls which you don’t want to receive.	925 (81.1%)	225 (74.3%)	454 (97.8%)	246 (65.8%)	151.25	0.36 ***
16. Following you.	885 (77.5%)	215 (70.7%)	450 (97.0%)	220 (58.8%)	183.80	0.40 ***
17. Taking photographs of you without your knowledge.	857 (75.0%)	167 (54.9%)	447 (96.3%)	243 (65.0%)	198.22	0.42 ***
18. Acting in an angry manner when seeing you out with other people (e.g., your friends or romantic partners).	855 (74.9%)	155 (50.8%)	433 (93.3%)	267 (71.4%)	178.50	0.40 ***
19. Intercepting mail/deliveries.	853 (74.8%)	184 (60.7%)	449 (96.8%)	220 (59.0%)	200.27	0.42 ***
Cluster 2: Unwanted attention (7 items)						
20. Sending you unwanted letters, notes, email, or other written communications.	850 (74.4%)	181 (59.5%)	447 (86.3%)	222 (59.4%)	197.06	0.42 ***
21. Refusing to accept that a prior relationship is over.	785 (68.8%)	143 (47.2%)	444 (95.7%)	198 (52.9%)	266.00	0.48 ***
22. Leaving unwanted items for you to find.	770 (67.4%)	121 (39.8%)	432 (93.1%)	217 (58.0%)	259.97	0.48 ***
23. Standing and waiting outside your home.	755 (66.2%)	177 (58.4%)	438 (94.4%)	140 (37.4%)	311.26	0.52 ***
24. Giving or sending you strange parcels.	736 (64.5%)	166 (54.8%)	448 (96.6%)	122 (32.6%)	386.69	0.58 ***
25. Standing and waiting outside your school or workplace.	648 (56.8%)	106 (35.0%)	431 (92.9%)	111 (29.7%)	417.14	0.60 ***
26. Driving, riding, or walking purposefully past your residence, school, or workplace.	636 (55.7%)	111 (36.6%)	434 (93.5%)	91 (24.3%)	463.04	0.64 ***
Cluster 3: Persistent courtship and impositions (9 items)						
27. Someone at a social event such as a party asks you if you would like to have sex with him/her.	998 (87.5%)	238 (78.3%)	445 (95.9%)	315 (84.5%)	56.59	0.22 ***
28. Someone engages you in an inappropriate personal and intimate discussion.	899 (78.7%)	188 (61.8%)	444 (95.7%)	267 (71.4%)	143.46	0.35 ***
29. “Wolf-whistling” in the street.	816 (71.5%)	175 (57.8%)	449 (96.8%)	192 (51.3%)	248.16	0.47 ***
30. Agreeing with your every word, even if you were wrong.	790 (69.2%)	131 (43.1%)	431 (92.9%)	228 (61.0%)	231.19	0.45 ***
31. “Outstaying his/her welcome” in your home.	783 (68.6%)	144 (47.5%)	439 (94.6%)	200 (53.5%)	248.05	0.47 ***
32. Asking you for a date repeatedly.	676 (59.2%)	120 (39.5%)	437 (94.2%)	119 (31.9%)	399.25	0.59 ***
33. A stranger offering to buy you a drink in a café, restaurant, or bar.	674 (59.0%)	122 (40.1%)	437 (94.2%)	115 (30.7%)	405.61	0.60 ***
34. Making arrangements without asking you first (e.g., booking a table at a restaurant).	656 (57.5%)	96 (31.7%)	434 (93.5%)	126 (33.7%)	415.93	0.60 ***
35. Sending or giving you gifts.	498 (43.6%)	26 (8.6%)	425 (91.6%)	47 (12.6%)	732.06	0.80 ***
Cluster 4: Courtship and information seeking (10 items)						
36. Doing unrequested favors for you.	640 (56.0%)	96 (31.6%)	452 (97.4%)	92 (24.6%)	546.33	0.69 ***
37. Talking about you to mutual friends after meeting you just once.	637 (55.8%)	62 (20.4%)	436 (94.0%)	139 (37.3%)	480.56	0.65 ***
38. Visiting places because she/he knows that you may be there.	626 (54.9%)	82 (27.0%)	425 (91.6%)	119 (31.9%)	427.70	0.61 ***
39. Changing classes, offices, or joining a new group to be closer to you.	582 (51.1%)	56 (18.5%)	432 (93.1%)	94 (25.2%)	556.72	0.70 ***
40. A stranger engaging you in a conversation in a public place (e.g., at a bus stop or in a café).	573 (50.2%)	70 (23.0%)	434 (93.5%)	69 (18.4%)	589.15	0.72 ***
41. Asking your friends, family, school, or work colleagues about you.	575 (50.1%)	58 (19.1%)	426 (91.8%)	88 (23.6%)	544.75	0.69 ***
42. Telephoning you after one initial meeting.	552 (48.3%)	48 (15.8%)	427 (92.0%)	77 (20.6%)	598.93	0.72 ***
43. Trying to get to know your friends in order to get to know you better.	547 (47.9%)	32 (10.6%)	427 (92.0%)	88 (23.5%)	620.26	0.74 ***
44. Seeing him/her at the same time each day.	539 (47.3%)	49 (16.2%)	426 (91.8%)	64 (17.2%)	622.54	0.74 ***
45. Asking you out “as just friends”.	487 (42.7%)	27 (8.9%)	429 (92.5%)	31 (8.3%)	791.99	0.83 ***
Cluster 5: Others (2 items)						
46. Coming round to visit you, uninvited, on a regular basis.	788 (69.1%)	131 (43.2%)	437 (94.2%)	220 (59.0%)	249.63	0.47 ***
47. Finding out information about you (phone numbers, marital status, address, hobbies) without asking you directly.	732 (64.2%)	133 (43.9%)	441 (95.0%)	158 (42.2%)	324.65	0.53 ***

*** *p* < 0.001.

**Table 2:** Geographical distribution of victimization experiences of stalking and intrusive behaviors (*n* = 1143).

Items	Victimization Experiences of Stalking and Intrusive Behaviors				Geographical Differences	
	Overall (*n* = 1143) *n* (%)	Hong Kong (*n* = 305) *n* (%)	Mainland China (*n* = 464) *n* (%)	Ghana (*n* = 374) *n* (%)	χ^2^	Cramer’s *V*
Cluster 1: Aggression and surveillance (19 items)						
01. Taking photographs of you without your knowledge.	519 (45.6%)	152 (50.8%)	117 (25.2%)	250 (66.8%)	149.05	0.36 ***
02. Verbally abusing you.	444 (39.1%)	82 (27.4%)	117 (25.2%)	245 (65.7%)	165.40	0.38 ***
03. Hurting you emotionally (verbal abuse, ruining your reputation).	444 (39.1%)	100 (33.4%)	130 (28.0%)	214 (57.2%)	79.55	0.26 ***
04. Confining you against your will.	438 (38.6%)	36 (12.0%)	99 (21.3%)	303 (81.7%)	437.64	0.62 ***
05. Secretly taking your belongings.	431 (37.9%)	57 (19.1%)	81 (17.5%)	293 (78.6%)	389.21	0.59 ***
06. Harming you physically.	419 (36.9%)	19 (6.4%)	67 (14.4%)	333 (89.3%)	659.93	0.76 ***
07. Criminal damage/vandalism to your property.	416 (36.6%)	18 (6.0%)	64 (13.8%)	334 (89.3%)	672.32	0.77 ***
08. Acting in an angry manner when seeing you out with other people (e.g., your friends or romantic partners).	417 (36.6%)	105 (35.0%)	118 (25.4%)	194 (51.9%)	62.83	0.23 ***
09. Making death threats.	414 (36.4%)	17 (5.7%)	69 (14.9%)	328 (87.7%)	639.82	0.75 ***
10. Intercepting mail/deliveries.	411 (36.1%)	22 (7.4%)	64 (13.8%)	325 (86.9%)	625.18	0.74 ***
11. Physically hurting someone you care about.	405 (35.6%)	14 (4.7%)	69 (14.9%)	322 (86.1%)	627.44	0.74 ***
12. Following you.	404 (35.5%)	76 (25.4%)	79 (17.0%)	249 (66.6%)	240.09	0.46 ***
13. Threatening to physically hurt you.	400 (35.2%)	12 (4.0%)	59 (12.7%)	329 (88.0%)	687.07	0.78 ***
14. Multiple telephone calls which you don’t want to receive.	388 (34.1%)	99 (33.1%)	111 (23.9%)	178 (47.6%)	51.80	0.21 ***
15. Trespassing on your property.	386 (34.0%)	14 (4.7%)	53 (11.4%)	319 (85.5%)	661.38	0.76 ***
16. Threatening to kill or hurt herself/himself if you refused to go out on a date with her/him.	383 (33.7%)	15 (5.0%)	76 (16.4%)	292 (78.1%)	502.12	0.66 ***
17. Forced sexual contact.	375 (33.0%)	13 (4.3%)	57 (12.3%)	305 (81.8%)	602.23	0.73 ***
18. Trying to manipulate or force you into dating her/him.	352 (31.0%)	30 (10.0%)	70 (15.1%)	252 (67.4%)	348.05	0.55 ***
19. Spying on you.	324 (28.5%)	38 (12.7%)	59 (12.7%)	227 (60.9%)	284.85	0.50 ***
Cluster 2: Unwanted attention (7 items)						
20. Sending you unwanted letters, notes, email, or other written communications.	476 (41.8%)	126 (42.0%)	144 (31.0%)	206 (55.1%)	49.21	0.21 ***
21. Leaving unwanted items for you to find.	399 (35.1%)	38 (12.7%)	53 (11.4%)	308 (82.4%)	546.64	0.69 ***
22. Standing and waiting outside your school or workplace.	395 (34.7%)	60 (20.1%)	44 (9.5%)	291 (77.8%)	464.93	0.64 ***
23. Refusing to accept that a prior relationship is over.	352 (31.0%)	55 (18.4%)	53 (11.4%)	244 (65.2%)	310.58	0.52 ***
24. Standing and waiting outside your home.	352 (31.0%)	49 (16.4%)	47 (10.1%)	256 (68.4%)	369.82	0.57 ***
25. Giving or sending you strange parcels.	352 (31.0%)	19 (6.4%)	38 (8.2%)	295 (78.9%)	599.00	0.73 ***
26. Driving, riding, or walking purposefully past your residence, school, or workplace.	351 (30.9%)	28 (9.4%)	41 (8.8%)	292 (75.4%)	517.88	0.67 ***
Cluster 3: Persistent courtship and impositions (9 items)						
27. Sending or giving you gifts.	481 (42.3%)	131 (43.8%)	125 (26.9%)	225 (60.2%)	94.01	0.29 ***
28. Agreeing with your every word, even if you were wrong.	440 (38.7%)	111 (37.0%)	117 (25.2%)	212 (56.7%)	86.95	0.28 ***
29. “Wolf-whistling” in the street.	433 (38.1%)	55 (18.4%)	56 (12.1%)	322 (86.1%)	547.96	0.69 ***
30. Asking you for a date repeatedly.	415 (36.5%)	89 (29.8%)	104 (22.4%)	222 (59.4%)	129.89	0.34 ***
31. Someone engages you in an inappropriate personal and intimate discussion.	390 (34.3%)	57 (19.1%)	108 (23.3%)	225 (60.2%)	166.81	0.38 ***
32. Someone at a social event such as a party asks you if you would like to have sex with him/her.	382 (33.6%)	26 (8.7%)	50 (10.8%)	306 (81.8%)	581.24	0.71 ***
33. Making arrangements without asking you first (e.g., booking a table at a restaurant).	375 (33.0%)	56 (18.7%)	59 (12.7%)	260 (69.5%)	339.57	0.55 ***
34. A stranger offering to buy you a drink in a café, restaurant, or bar.	337 (29.6%)	57 (19.1%)	43 (9.3%)	237 (63.4%)	312.41	0.52 ***
35. “Outstaying his/her welcome” in your home.	317 (27.9%)	18 (6.0%)	46 (9.9%)	253 (67.6%)	439.69	0.62 ***
Cluster 4: Courtship and information seeking (10 items)						
36. Doing unrequested favors for you.	479 (42.1%)	124 (41.5%)	150 (32.3%)	205 (54.8%)	43.02	0.19 ***
37. Asking your friends, family, school, or work colleagues about you.	467 (41.1%)	163 (54.5%)	131 (28.2%)	173 (46.4%)	58.26	0.23 ***
38. Talking about you to mutual friends after meeting you just once.	452 (39.8%)	133 (44.5%)	125 (26.9%)	194 (52.0%)	57.98	0.23 ***
39. A stranger engaging you in a conversation in a public place (e.g., at a bus stop or in a café).	453 (39.8%)	217 (72.3%)	129 (27.8%)	107 (28.6%)	179.94	0.40 ***
40. Trying to get to know your friends in order to get to know you better.	436 (38.3%)	116 (38.8%)	120 (25.9%)	200 (53.5%)	66.83	0.24 ***
41. Visiting places because she/he knows that you may be there.	421 (37.0%)	82 (27.4%)	96 (20.7%)	243 (65.0%)	190.21	0.41 ***
42. Changing classes, offices, or joining a new group to be closer to you.	413 (36.3%)	57 (19.1%)	62 (13.4%)	294 (78.6%)	433.41	0.62 ***
43. Asking you out “as just friends”.	408 (35.9%)	116 (38.8%)	96 (20.7%)	196 (52.4%)	92.04	0.28 ***
44. Seeing him/her at the same time each day.	398 (35.0%)	57 (19.1%)	58 (12.5%)	283 (75.7%)	408.51	0.60 ***
45. Telephoning you after one initial meeting.	363 (31.9%)	96 (32.1%)	90 (19.4%)	177 (47.3%)	74.33	0.26 ***
Cluster 5: Others (2 items)						
46. Finding out information about you (phone numbers, marital status, address, hobbies) without asking you directly.	411 (36.1%)	149 (49.8%)	86 (18.5%)	176 (47.1%)	105.92	0.31 ***
47. Coming round to visit you, uninvited, on a regular basis.	344 (30.3%)	39 (13.0%)	71 (15.3%)	234 (62.6%)	276.19	0.49 ***

*** *p* < 0.001.

**Table 3:** Geographical distribution of perceived worst experiences of stalking and intrusive behavior victimization frequency (*n* = 1143).

Items	Frequency of Perceived Worst Victimization Experience of Stalking and Intrusive Behaviors								Geographical Differences	
	Overall		Hong Kong		Mainland China		Ghana			
	1–4 Times *n* (%)	5+ Times *n* (%)	1–4 Times *n* (%)	5+ Times *n* (%)	1–4 Times *n* (%)	5+ Times *n* (%)	1–4 Times *n* (%)	5+ Times *n* (%)	χ^2^	Cramer’s *V*
01. Phone calls, text messages, gifts, or letter.	376 (39.6%)	302 (31.8%)	107 (37.9%)	79 (28.0%)	166 (50.2%)	74 (22.4%)	103 (30.6%)	149 (44.2%)	47.75	0.16 ***
02. Emails and/or messages on social media (e.g., Facebook) and other web-based communications.	300 (33.4%)	209 (23.3%)	111 (39.4%)	48 (17.0%)	118 (37.2%)	80 (25.2%)	71 (23.7%)	81 (27.1%)	24.67	0.12 ***
03. Trespassing on your property.	112 (12.5%)	37 (4.1%)	15 (5.4%)	1 (0.4%)	55 (16.6%)	24 (7.2%)	42 (14.6%)	12 (4.2%)	40.56	0.15 ***
04. Damage to your property, wrecking things you care about, or hurting your pet.	108 (12.1%)	35 (3.9%)	20 (7.1%)	5 (1.8%)	59 (18.2%)	26 (8.0%)	29 (10.2%)	4 (1.4%)	44.27	0.16 ***
05. Surveillance (following you, watching you, recording you).	199 (22.3%)	71 (8.0%)	66 (23.4%)	8 (2.8%)	49 (15.6%)	28 (8.9%)	84 (28.3%)	35 (11.8%)	32.60	0.14 ***
06. Ruining your reputation (sharing private pictures of you or sharing information about you, spreading lies about you).	213 (23.2%)	90 (9.8%)	65 (23.0%)	18 (6.4%)	84 (25.8%)	41 (12.6%)	64 (20.6%)	31 (10.0%)	10.02	0.07 *
07. Threatening to hurt you.	129 (14.4%)	31 (3.5%)	20 (7.1%)	2 (0.7%)	58 (17.9%)	20 (6.2%)	51 (17.5%)	9 (3.1%)	33.46	0.14 ***
08. Threatening to hurt people you care about.	103 (11.6%)	36 (4.1%)	18 (6.4%)	1 (0.4%)	45 (14.0%)	27 (8.4%)	40 (14.0%)	8 (2.8%)	39.25	0.15 ***
09. Actually hurting people you care about.	100 (11.2%)	31 (3.5%)	11 (3.9%)	3 (1.1%)	51 (15.9%)	19 (5.9%)	38 (13.2%)	9 (3.1%)	36.13	0.14 ***
10. Physically and/or sexually attacking you.	103 (11.6%)	43 (4.8%)	17 (6.0%)	3 (1.1%)	41 (12.8%)	23 (7.2%)	45 (15.5%)	17 (5.9%)	28.56	0.13 ***
11. Threatening to or actually hurting him/herself.	151 (17.0%)	42 (4.7%)	25 (9.0%)	2 (0.7%)	56 (17.2%)	27 (8.3%)	70 (24.6%)	13 (4.6%)	46.24	0.16 ***
12. Harassing other people to upset you or find out information about you.	182 (20.3%)	70 (7.8%)	51 (18.0%)	8 (2.8%)	80 (24.8%)	32 (9.9%)	51 (17.5%)	30 (10.3%)	22.36	0.11 ***
13. Forcing you to talk to him/her.	264 (29.0%)	112 (12.3%)	82 (29.2%)	19 (6.8%)	81 (25.5%)	30 (9.4%)	101 (32.4%)	63 (20.2%)	37.86	0.14 ***
14. Declaring love for you.	301 (32.7%)	211 (22.9%)	91 (32.4%)	25 (8.9%)	137 (43.2%)	60 (18.9%)	73 (22.6%)	126 (39.0%)	102.30	0.24 ***
15. Getting other people to engage in any of the 14 behaviors listed above on their behalf.	154 (17.1%)	54 (6.0%)	31 (11.2%)	2 (0.7%)	52 (16.0%)	25 (7.7%)	71 (24.1%)	27 (9.2%)	42.16	0.15 ***

* *p* < 0.05, *** *p* < 0.001.

**Table 4:** Geographical distribution of perceived worst victimization experience of stalking and intrusive behavior duration (*n* = 1143).

Items	Duration of Perceived Worst Victimization Experience of Stalking and Intrusive Behavior												Geographical Differences	
	Overall			Hong Kong			Mainland China			Ghana				
	<2 Weeks *n* (%)	2 Weeks–6 Moths *n* (%)	>6 Months *n* (%)	<2 Weeks *n* (%)	2 Weeks–6 Moths *n* (%)	>6 Months *n* (%)	<2 Weeks *n* (%)	2 Weeks–6 Moths *n* (%)	>6 Months *n* (%)	<2 Weeks *n* (%)	2 Weeks–6 Moths *n* (%)	>6 Months *n* (%)	χ^2^	Cramer’s *V*
01. Phone calls, text messages, gifts, or letter.	255 (37.1%)	231 (33.6%)	201 (29.3%)	96 (51.6%)	66 (35.5%)	24 (12.9%)	94 (50.3%)	63 (33.7%)	30 (16.0%)	65 (20.7%)	102 (32.5%)	147 (46.8%)	103.60	0.27 ***
02. Emails and/or messages on social media (e.g., Facebook) and other web-based communications.	281 (47.6%)	177 (30.0%)	132 (22.4%)	77 (49.4%)	55 (35.3%)	24 (15.4%)	89 (53.6%)	51 (30.7%)	26 (15.7%)	115 (42.9%)	71 (26.5%)	82 (30.6%)	20.01	0.13 ***
03. Trespassing on your property.	277 (75.5%)	68 (18.5%)	22 (6.0%)	8 (47.1%)	7 (41.2%)	2 (11.8%)	70 (72.9%)	19 (19.8%)	7 (7.3%)	199 (78.3%)	42 (16.5%)	13 (5.1%)	9.05	0.11 ^+^
04. Damage to your property, wrecking things you care about, or hurting your pet.	296 (79.6%)	56 (15.1%)	20 (5.4%)	15 (68.2%)	3 (13.6%)	4 (18.2%)	65 (66.3%)	23 (23.5%)	10 (10.2%)	216 (85.7%)	30 (11.9%)	6 (2.4%)	25.18	0.18 ***
05. Surveillance (following you, watching you, recording you).	260 (58.4%)	126 (28.3%)	59 (13.3%)	40 (54.8%)	21 (28.8%)	12 (16.4%)	75 (72.1%)	19 (18.3%)	10 (9.6%)	145 (54.1%)	86 (32.1%)	37 (13.8%)	11.08	0.11 *
06. Ruining your reputation (sharing private pictures of you or sharing information about you, spreading lies about you).	295 (62.4%)	118 (24.9%)	60 (12.7%)	45 (55.6%)	21 (25.9%)	15 (18.5%)	70 (60.9%)	33 (28.7%)	12 (10.4%)	180 (65.0%)	64 (23.1%)	33 (11.9%)	4.77	0.07
07. Threatening to hurt you.	275 (72.8%)	82 (21.7%)	21 (5.6%)	12 (54.5%)	8 (36.4%)	2 (9.1%)	66 (67.3%)	23 (23.5%)	9 (9.2%)	197 (76.4%)	51 (19.8%)	10 (3.9%)	8.75	0.11 ^+^
08. Threatening to hurt people you care about.	272 (75.6%)	67 (18.6%)	21 (5.8%)	8 (47.1%)	9 (52.9%)	0 (0.0%)	61 (67.8%)	18 (20.0%)	11 (12.2%)	203 (80.2%)	40 (15.8%)	10 (4.0%)	20.03	0.18 ***
09. Actually hurting people you care about.	285 (77.4%)	60 (16.3%)	23 (6.3%)	4 (30.8%)	6 (46.2%)	3 (23.1%)	76 (75.2%)	14 (13.9%)	11 (10.9%)	205 (80.7%)	40 (15.7%)	9 (3.5%)	23.94	0.18 ***
10. Physically and/or sexually attacking you.	261 (71.3%)	71 (19.4%)	34 (9.3%)	11 (55.0%)	4 (20.0%)	5 (25.0%)	53 (61.6%)	22 (25.6%)	11 (12.8%)	197 (75.8%)	45 (17.3%)	18 (6.9%)	12.90	0.13 *
11. Threatening to or actually hurting him/herself.	248 (66.8%)	97 (26.1%)	26 (7.0%)	16 (57.1%)	8 (28.6%)	4 (14.3%)	65 (71.4%)	18 (19.8%)	8 (8.8%)	167 (66.3%)	71 (28.2%)	14 (5.6%)	5.85	0.09
12. Harassing other people to upset you or find out information about you.	268 (65.5%)	94 (23.0%)	47 (11.5%)	32 (56.1%)	17 (29.8%)	8 (14.0%)	62 (65.3%)	24 (25.3%)	9 (9.5%)	174 (67.7%)	53 (20.6%)	30 (11.7%)	3.62	0.07
13. Forcing you to talk to him/her.	231 (47.9%)	162 (33.6%)	89 (18.5%)	53 (53.5%)	32 (32.3%)	14 (14.1%)	63 (60.6%)	30 (28.8%)	11 (10.6%)	115 (41.2%)	100 (35.8%)	64 (22.9%)	15.44	0.13 **
14. Declaring love for you.	210 (37.5%)	188 (33.6%)	162 (28.9%)	42 (37.8%)	52 (46.8%)	17 (15.3%)	78 (49.1%)	63 (39.6%)	18 (11.3%)	90 (31.0%)	73 (25.2%)	127 (43.8%)	68.86	0.25 ***
15. Getting other people to engage in any of the 14 behaviors listed above on their behalf.	246 (62.6%)	106 (27.0%)	41 (10.4%)	17 (50.0%)	14 (41.2%)	3 (8.8%)	61 (65.6%)	20 (21.5%)	12 (12.9%)	168 (63.2%)	72 (27.1%)	26 (9.8%)	5.32	0.08

^+^*p* < 0.10, * *p* < 0.05, ** *p* < 0.01, *** *p* < 0.001.

**Table 5:** Geographical distribution of perceptions and victimization experiences of stalking and intrusive behaviors (*n* = 1143).

Items	Perceptions and Victimization Experiences of Stalking and Intrusive Behaviors							
	Overall (*n* = 1143)		Hong Kong (*n* = 305)		Mainland China (*n* = 464)		Ghana (*n* = 374)	
	Perceptions %	Experiences %	Perceptions %	Experiences %	Perceptions %	Experiences %	Perceptions %	Experiences %
Cluster 1: Aggression and surveillance (19 items)								
01. Making death threats.	94.0%	36.4%	93.1%	5.7%	97.4%	14.9%	90.6%	87.7%
02. Criminal damage/vandalism to your property.	92.6%	36.6%	90.1%	6.0%	96.8%	13.8%	89.5%	89.3%
03. Threatening to kill or hurt herself/himself if you refused to go out on a date with her/him.	92.0%	33.7%	92.1%	5.0%	97.4%	16.4%	85.3%	78.1%
04. Forced sexual contact.	90.8%	33.0%	91.7%	4.3%	97.8%	12.3%	81.2%	81.8%
05. Harming you physically.	90.7%	36.9%	90.8%	6.4%	96.8%	14.4%	83.2%	89.3%
06. Verbally abusing you.	90.6%	39.1%	84.5%	27.4%	97.8%	25.2%	86.6%	65.7%
07. Threatening to physically hurt you.	89.4%	35.2%	88.4%	4.0%	97.2%	12.7%	80.4%	88.0%
08. Hurting you emotionally (verbal abuse, ruining your reputation).	88.2%	39.1%	80.5%	33.4%	97.0%	28.0%	83.4%	57.2%
09. Physically hurting someone you care about.	87.2%	35.6%	83.5%	4.7%	96.8%	14.9%	78.3%	86.1%
10. Secretly taking your belongings.	85.8%	37.9%	80.9%	39.3%	98.3%	17.5%	75.7%	78.6%
11. Trying to manipulate or force you into dating her/him.	84.8%	31.0%	81.5%	10.0%	97.2%	15.1%	71.9%	67.4%
12. Spying on you.	84.1%	28.5%	84.2%	12.7%	97.6%	12.7%	67.4%	60.9%
13. Trespassing on your property.	83.0%	34.0%	73.6%	4.7%	97.0%	11.4%	73.3%	85.5%
14. Confining you against your will.	82.6%	38.6%	68.6%	12.0%	97.2%	21.3%	75.9%	81.7%
15. Multiple telephone calls which you don’t want to receive.	81.1%	34.1%	74.3%	33.1%	97.8%	23.9%	65.8%	47.6%
16. Following you.	77.5%	35.5%	70.7%	25.4%	97.0%	17.0%	58.8%	66.6%
17. Taking photographs of you without your knowledge.	75.0%	45.6%	54.9%	50.8%	96.3%	25.2%	65.0%	66.8%
18. Acting in an angry manner when seeing you out with other people (e.g., your friends or romantic partners).	74.9%	36.6%	51.0%	35.0%	93.3%	25.4%	71.4%	51.9%
19. Intercepting mail/deliveries.	74.8%	36.1%	60.7%	7.4%	96.8%	13.8%	59.0%	86.9%
Cluster 2: Unwanted attention (7 items)								
20. Sending you unwanted letters, notes, email, or other written communications.	74.4%	41.8%	59.5%	42.0%	96.3%	31.0%	59.4%	55.1%
21. Refusing to accept that a prior relationship is over.	68.8%	31.0%	47.2%	18.4%	95.7%	11.4%	52.9%	65.2%
22. Leaving unwanted items for you to find.	67.4%	35.1%	39.8%	12.7%	93.1%	11.4%	58.0%	82.4%
23. Standing and waiting outside your home.	66.2%	31.0%	58.4%	16.4%	94.4%	10.1%	37.4%	68.4%
24. Giving or sending you strange parcels.	64.5%	31.0%	54.8%	6.4%	96.6%	8.2%	32.6%	78.9%
25. Standing and waiting outside your school or workplace.	56.8%	34.7%	35.0%	20.1%	92.9%	9.5%	29.7%	77.8%
26. Driving, riding, or walking purposefully past your residence, school, or workplace.	55.7%	30.9%	36.6%	9.4%	93.5%	8.8%	24.3%	75.4%
Cluster 3: Persistent courtship and impositions (9 items)								
27. Someone at a social event such as a party asks you if you would like to have sex with him/her.	87.5%	33.6%	78.3%	8.7%	95.9%	10.8%	84.5%	81.8%
28. Someone engages you in an inappropriate personal and intimate discussion.	78.7%	34.3%	61.8%	19.1%	95.7%	23.3%	71.4%	60.2%
29. “Wolf-whistling” in the street.	71.5%	38.1%	57.8%	18.4%	96.8%	12.1%	21.1%	48.7%
30. Agreeing with your every word, even if you were wrong.	69.2%	38.7%	43.1%	37.0%	92.9%	25.2%	61.0%	56.7%
31. “Outstaying his/her welcome” in your home.	68.6%	27.9%	47.5%	6.0%	94.6%	9.9%	53.5%	67.6%
32. Asking you for a date repeatedly.	59.2%	36.5%	39.5%	29.8%	94.2%	22.4%	31.9%	59.4%
33. A stranger offering to buy you a drink in a café, restaurant, or bar.	59.0%	29.6%	40.1%	19.1%	94.2%	9.3%	30.7%	63.4%
34. Making arrangements without asking you first (e.g., booking a table at a restaurant).	57.5%	33.0%	31.7%	18.7%	93.5%	12.7%	33.7%	69.5%
35. Sending or giving you gifts.	43.6%	42.3%	8.6%	43.8%	91.6%	26.9%	12.6%	60.2%
Cluster 4: Courtship and information seeking (10 items)								
36. Doing unrequested favors for you.	56.0%	42.1%	31.6%	41.5%	97.4%	32.3%	24.6%	54.8%
37. Talking about you to mutual friends after meeting you just once.	55.8%	39.8%	20.4%	44.5%	94.0%	26.9%	37.3%	52.0%
38. Visiting places because she/he knows that you may be there.	54.9%	37.0%	27.0%	27.4%	91.6%	20.7%	31.9%	65.0%
39. Changing classes, offices, or joining a new group to be closer to you.	51.1%	36.3%	18.5%	19.1%	93.1%	13.4%	25.2%	78.6%
40. A stranger engaging you in a conversation in a public place (e.g., at a bus stop or in a café).	50.2%	39.8%	23.0%	72.3%	93.5%	27.8%	18.4%	28.6%
41. Asking your friends, family, school, or work colleagues about you.	50.1%	41.1%	19.1%	54.5%	91.8%	28.2%	23.6%	46.4%
42. Telephoning you after one initial meeting.	48.3%	31.9%	15.8%	32.1%	92.0%	19.4%	20.6%	47.3%
43. Trying to get to know your friends in order to get to know you better.	47.9%	38.3%	10.6%	38.8%	92.0%	25.9%	23.5%	53.5%
44. Seeing him/her at the same time each day.	47.3%	35.0%	83.6%	19.1%	91.8%	12.5%	17.2%	75.7%
45. Asking you out “as just friends”.	42.7%	35.9%	8.9%	38.8%	92.5%	20.7%	8.3%	52.4%
Cluster 5: Others (2 items)								
46. Coming round to visit you, uninvited, on a regular basis.	69.1%	30.3%	43.2%	13.0%	94.2%	15.3%	59.0%	62.6%
47. Finding out information about you (phone numbers, marital status, address, hobbies) without asking you directly.	64.2%	36.1%	43.9%	49.8%	95.0%	18.5%	42.2%	47.1%

## Data Availability

The data presented in this study are available on request from the corresponding author.
